# Risk of SARS-CoV-2 Infection Among Essential Workers in a Community-Based Cohort in the United States

**DOI:** 10.3389/fpubh.2022.878208

**Published:** 2022-05-17

**Authors:** Chih-Fu Wei, Fan-Yun Lan, Yu-Tien Hsu, Nina Lowery, Lauren Dibona, Ream Akkeh, Stefanos N. Kales, Justin Yang

**Affiliations:** ^1^Department of Environmental Health, Harvard University T.H. Chan School of Public Health, Boston, MA, United States; ^2^Department of Occupational and Environmental Medicine, National Cheng Kung University Hospital, College of Medicine, National Cheng Kung University, Tainan, Taiwan; ^3^Department of Occupational Medicine, Cambridge Health Alliance, Harvard Medical School, Cambridge, MA, United States; ^4^Department of Social and Behavioral Science, Harvard University T.H. Chan School of Public Health, Boston, MA, United States; ^5^Manet Community Health Center, Quincy, MA, United States; ^6^Department of Employee and Occupational Health, Atrius Health, Boston, MA, United States; ^7^Department of General Internal Medicine, Boston University School of Medicine, Boston, MA, United States

**Keywords:** COVID-19, communicable diseases, occupational health, healthcare workers, Public Health Surveillance

## Abstract

**Objectives:**

The objective of this paper is to identify the risk factors for SARS-CoV-2 infection that are related to occupation type as well as workplace conditions. Identifying such risk factors could have noteworthy implications in workplace safety enhancement and emergency preparedness planning for essential workers.

**Methods:**

We conducted a retrospective analysis of visits at a community-based SARS-CoV-2 testing site in the greater Boston area between March 18^th^ and June 19^th^, 2020, for individuals between 14 and 65 years of age. Nasopharyngeal swab specimen, medical review, and self-administered questionnaire were obtained, and SARS-CoV-2 infection was determined with real-time, reverse transcriptase-polymerase chain reaction (RT-PCR). Medical record-verified job classification, customer-facing, and work patterns were extracted from each individual's response through chart review and validated by licensed clinicians. The occupational patterns were coded by occupational medicine physicians with pre-specified criteria and were analyzed with logistic regression and inverse probability weighting.

**Results:**

Among the 780 individuals included in the final analysis, working in healthcare-related jobs was associated with a four-fold increase in risk of SARS-CoV-2 infection (Adjusted OR: 4.00, 95% CI: 1.45–11.02). Individuals with customer-facing jobs had a two times risk increase (Adjusted OR: 1.97, 95% CI: 1.12–3.45) in having a positive SARS-CoV-2 RT-PCR assay result compared to participants with non-customer facing positions.

**Conclusions:**

In this U.S. community-based population during the initial wave of the pandemic, a significant increase in risk of SARS-CoV-2 infection was observed in those employed in the healthcare sector or with customer-facing positions. Further research is warranted to determine if these correlations continued with the buildup of population immunity together with the attenuation of SARS-CoV-2 virulence.

## Introduction

The Coronavirus disease 2019 (COVID-19) pandemic has become one of the worst pandemics in this century which has affected billions of people around the world since late 2019 (1, 2). Severe acute respiratory syndrome coronavirus 2 (SARS-CoV-2), the virus causing the COVID-19 pandemic, is transmitted via aerosol and droplets (3, 4) and has a longer survival duration that potentiated the transmission capacity (5). Several drastic public health interventions were implemented around the world during the initial phase of the pandemic, such as business closures, city-wide lockdowns, and stay-at-home orders, which created significant socioeconomic impact on the society (6–8). Meanwhile, population health measures such as universal masking and social distancing were effective interventions to slow down the spread of COVID-19. The development and availability of the COVID-19 vaccines and pharmacological treatments further reduced the risk of severe illnesses and deaths while the virus continues to attenuate to less virulent variants (9).

Throughout the pandemic, workers are subjected to these constant, often drastic, societal changes as continued commerce activities are indispensable to our society. Therefore, occupational health has been an integral part of the disease prevention discussions since the onset of this pandemic. The discussion ranged from the early days of protecting essential workers to ensure the continuance of critical operations during the first wave, to the recent concerns of reopening businesses safely under this “new normal” (10–13). Understanding the associations between work conditions, work-related exposure risks and SARS-CoV-2 infection may support guidance and recommendations ranging from workplace environment modifications to targeted surveillance among workers with higher infection risk (14). Workplace preventive interventions could significantly impact the society, reduce the transmission of pathogen at work, and protect the population at large (12, 15–20).

Healthcare workers (HCWs) have historically been the research focus for occupational health as they work within an environment with significantly higher and uncertain exposure risks (21). Study in 11 Midwestern U.S. states found healthcare workers had a four-fold increase in risk of filing COVID-19 related Workers' compensation claims (22). Various studies throughout the pandemic have focused on the work conditions for healthcare workers, such as the proper use of personal protective equipment (PPE) was frequently associated with a decreased risk in SARS-CoV-2 infection among HCWs (23–25).

At the same time, work-related risks for SARS-CoV-2 infection among non-healthcare essential workers in the community continue to remain unclear even as businesses have largely reopened and as the society continued to adjust to different phases of this pandemic (12, 18–20, 26). Our study published early in the pandemic observed significant work-related transmission in service workers and drivers with COVID-19 exposure history in six Asian countries (19). In the U.S., only limited, industry-specific reports and studies provided some insights on non-HCW occupational exposure risks, such as the outbreak in meat-processing factories that identified congregated work and residential locations as risk factors, and the grocery store outbreak in Massachusetts that suggested customer contact as a risk factor for retail workers (12, 18, 20, 26). No study to-date has examined how job categories, occupations and customer-facing conditions influence SARS-CoV-2 infection risk at a community level in the U.S. Therefore, in this study we aim to examine the associations between job categories, occupational exposure, and SARS-CoV-2 test results among a cohort of community residents during the initial wave of the pandemic by utilizing occupational health physician-verified job categories, customer-facing conditions, and SARS-CoV-2 real-time, reverse transcriptase-polymerase chain reaction (RT-PCR) assay results, adjusting for known socio-behavioral confounders (27). We hypothesized that both job categories and customer-facing conditions impact a worker's risk of contracting SARS-CoV-2 infection after controlling for covariates.

## Materials and Methods

### Study Population Selection and Setting

The study population was based on data from a city-supported COVID-19 testing clinic in Quincy, Massachusetts, which provided no-cost clinical evaluation and testing for the general population in the community with suspected COVID-19 related symptoms, contact, or travel exposure.

Our study included individuals aged 18 and above who presented for a clinical evaluation and received SARS-CoV-2 RT-PCR testing during the study period between March 18 and June 19 in 2020. Additionally, we included individuals between the age of 14–18 who indicated a current employment status to capture minors working part-time during the pandemic. We excluded patients tested for (1) State-sponsored post-mass-gathering/ protest testing initiative, (2) mandatory contact tracing testing events for homeless shelters and private institutions, and (3) retests after SARS-CoV-2 infection. We particularly selected the study period between March 18, 2020, and June 19, 2020, which reflected the first wave of coronavirus pandemic in the study region (28, 29).

### Data Collection and Quality Control

We extracted baseline demographic information (name, age, gender, and race/ethnicity), day of the clinic visit, and SARS-CoV-2 RT-PCR test results from a database established by the clinic's data analyst. At the time the participants got COVID-19 testing, their information (sociodemographic and occupational history) was recorded by the clinic's staff. We then cross-referenced the list with the clinic's electronic medical record system, reviewed and extracted relevant information from the templated telemedicine clinical notes recorded by licensed clinicians and electronic intake forms from patients entered on an iPad prior to receiving SARS-CoV-2 testing. We also reviewed and validated medical charts for the individual's presenting clinical symptoms, date of symptom onset (if with symptoms), SARS-CoV-2 exposure history (if any), current occupation/ job title and last day of work, recent travel history, household population, and smoking status. The clinical symptoms in this study included fever, headache, cough, shortness of breath, sore throat, myalgia, fatigue, nausea/vomiting, diarrhea, and anosmia. The chart review process was equally and randomly assigned to three licensed clinicians (NL, LD, and RA) by their clinic visit date. The chart reviewers discussed any unclear or uncertain situations within the group and with JY, and final extraction decisions were then made by JY after discussions. To ensure chart review quality, a total of 20 charts were selected randomly and reviewed by JY from each chart reviewer. The database was then deidentified prior to further review and statistical analysis.

### Definition of Job and Work-Related Conditions

We included job classification, customer-facing, interval since last day at work, and work patterns (not at work, work from home, or in person) in this study. We extracted the individual's current work status directly from the medical records as a three-leveled response (“no,” “yes”, and “yes but work from home”). We further extracted their last date at work if a date was given by the individual during intake. Meanwhile, we categorized job classification and customer-facing conditions by independent clinician review followed by a panel discussion for all individuals who provided their job information during the initial intake. Specifically, three occupational medicine physicians (CFW, FYL, YTH) independently reviewed the job titles from the deidentified database and determined the initial coding for job category and customer-facing conditions. The job family of each patient was defined by matching each individual's self-reported job to the closest job families listed in O^*^NET OnLine, a U.S. Department of Labor-sponsored database (30). The three physicians coded customer-facing conditions at work as “yes” or “no”, based on their likelihood of customer facing conditions for given job titles as determined by the reviewer. Then, a consensus of job classification and customer exposure was reached for each patient by combining and comparing independent category coding conducted by CFW, FYL, YTH. Any discrepancies were discussed together was a group and with JY for a final decision. For individuals with uncertain job category or customer exposure status after discussions, JY would conduct follow-up telephone for further clarification by the patients. Final coding for each patient was reexamined by all the discussants in the final discussion round, after resolving any residual discrepancy or possible misspecification (CFW, FYL, YTH, and JY).

### SARS-CoV-2 Testing and Specimen Collection

Trained clinician obtained nasopharyngeal specimens from individuals and stored them in a 3 ml vial with viral transport media (VTM). The samples were transported on ice to Quest Diagnostic laboratory in Marlborough, Massachusetts for RT-PCR analysis. The collection process followed guidelines published by the U.S. Center for Disease Control and Prevention (CDC) (31). Patients' SARS-CoV-2 assay result was reported as positive, negative, or indeterminate (32).

### Definition of Confounders

The confounders were selected based upon available literature on SARS-CoV-2 infection and COVID-19 (18, 23, 24, 27, 33–39). We manually extracted age, gender, race, smoking status, household population size, travel history, and self-reported contact from each medical record. Race and ethnicity were grouped into non-Hispanic white, non-Hispanic black, Asian, Hispanic, and others. Smoking condition and travel history were dichotomized into binary variables (yes or no). Self-reported contact history was categorized as no, yes (with family members or friends), and yes (with colleagues or customers). We defined an interval indicator as to the date of testing eligibility expansion at the study site (April 19, 2020) and the initiation of Phase 1 reopening in Massachusetts (May 18, 2020) (28).

### Statistical Analysis

Continuous variables were inspected for normality with a Q-Q plot first. Then, these continuous variables were presented in their means and standard deviations among the population with positive and negative results, respectively. Meanwhile, categorical variables were presented in count and percentage. *P*-values were tested with independent *t*-test for continuous variables and were tested using χ^2^ or Fisher exact test for categorical ones. The percentages were presented in rows to highlight the proportion of positive and negative tests for each level of the variables. We applied multivariable logistic regression models to examine the association between the primary outcome of positive SARS-CoV-2 RT-PCR assays and different work conditions. We demonstrated both unadjusted, adjusted odds ratio (OR) and 95% confidence intervals (CI) adjusted for all confounders listed above. We let people not currently working or working from home be the reference group for the association between job categories, and we set the non-exposed individuals as the reference group for the association of customer-facing, contact the source and work from home status.

The dataset was extracted and reviewed in Microsoft Excel, and analyses were performed using the R software, version 4.0.4. All *p*-values are two-tailed and without adjustment for multiple testing, and we used a significance level of 0.05 in this study.

### Sensitivity Analysis

We tested the associations in the multivariable regression model adjusting for all other non-occupational factors, which captures the association between known risk factors and the risk of SARS-CoV-2 infection. Then, we examined the association between SARS-CoV-2 assay results and job categories, work status, and customer-facing exposure for patients presented before the date of Massachusetts Phase 1 reopening. This subpopulation is more reflective of essential workers and is indicative of the population at risk during the first wave of the pandemic (28). Furthermore, we applied inverse probability weighting to balance the covariate distribution in the whole population, in which we balanced the probability of being in each work groups with their symptoms at presentation. So, the association between different job categories was not confounded by indication of testing. We presented demographic characteristics in different work statuses, and clinical symptoms at their baseline visits. Lastly, we demonstrated the clinical and household conditions for work-from-home individuals who tested positive for SARS-CoV-2.

### Human Subjects

All medical records and test results were de-identified at the primary clinical site. The de-identified database was then transferred by secure email system to Harvard TH Chan School of Public Health for analysis. The study of de-identified data received a non-human research exempt determination by the Institutional Review Board of Boston University (IRB H-40496).

## Results

### Characteristics of the Study Population

A total of 2,257 patients received testing at this clinic location during the study period between March 18 and June 19 in 2020. We included 780 individuals that met our selection criteria in the final analyses, with 95 of them (12.2%) testing positive for SARS-CoV-2 by RT-PCR assay. The mean age of the study population was 42.0 years old (SD: 12.7 years); the majority of the participants were female (56.9%) and non-Hispanic Caucasians (63.7%) (Table 1). There were 190 current smokers (24.4%) in the study population. Self-reported COVID-19 exposure history were mentioned among 313 individuals (147 from families and friends, and 166 from colleague and customer), and only 44 subjects in the study population reported travel history during the study period.

**Table 1:** Comparison of baseline sociodemographic, job category, and work condition in study population between March 18,2020 and June 19, 2020, stratified by SARS-CoV-2 RT-PCR assay results^a^.

	**Overall**	**Positive**	**Negative**	***p*** **-value**
*N* (%)	780 (100.0%)	95 (12.2%)	685 (87.8%)	
Age (mean (SD))	42.0 (12.7)	40.7 (14.2)	42.1 (12.4)	0.288
Gender (%)				0.782
Female	443 (100.0%)	52 (11.7%)	391 (88.3%)	
Male	335 (100.0%)	43 (12.8%)	292 (87.2%)	
Race (%)				<0.001
Non-Hispanic white	443 (100.0%)	30 (6.8%)	413 (93.2%)	
Black	56 (100.0%)	16 (28.6%)	40 (71.4%)	
Asian	77 (100.0%)	13 (16.9%)	64 (83.1%)	
Hispanics	44 (100.0%)	10 (22.7%)	34 (77.3%)	
Others	75 (100.0%)	16 (21.3%)	59 (78.7%)	
Smoking (%)				< 0.001
No	589 (100.0%)	87 (14.8%)	502 (85.2%)	
Yes	190 (100.0%)	8 (4.2%)	182 (95.8%)	
Household population size (mean (SD))	3.1 (1.8)	3.5 (1.9)	3.0 (1.8)	0.012
Contact history (%)				< 0.001
No	464 (100.0%)	41 (8.8%)	423 (91.2%)	
Family/Friend	147 (100.0%)	37 (25.2%)	110 (74.8%)	
Colleague/Customer	166 (100.0%)	17 (10.2%)	149 (89.8%)	
Travel history (%)				0.012
No	734 (100.0%)	93 (12.7%)	641 (87.3%)	
Yes	44 (100.0%)	0 (0.0%)	44 (100.0%)	
Job families (%)				0.749
Not working	324 (100.0%)	41 (12.7%)	283 (87.3%)	
Architecture and engineering	3 (100.0%)	0 (0.0%)	3 (100.0%)	
Building and grounds cleaning and maintenance	15 (100.0%)	2 (13.3%)	13 (86.7%)	
Business and financial operations	5 (100.0%)	0 (0.0%)	5 (100.0%)	
Community and social service	29 (100.0%)	3 (10.3%)	26 (89.7%)	
Computer and mathematical	1 (100.0%)	0 (0.0%)	1 (100.0%)	
Construction and extraction	34 (100.0%)	2 (5.9%)	32 (94.1%)	
Education, training, and library	4 (100.0%)	0 (0.0%)	4 (100.0%)	
Food preparation and serving	44 (100.0%)	7 (15.9%)	37 (84.1%)	
Healthcare practitioners and technical	40 (100.0%)	10 (25.0%)	30 (75.0%)	
Healthcare support	23 (100.0%)	2 (8.7%)	21 (91.3%)	
Installation, maintenance, and repair	12 (100.0%)	0 (0.0%)	12 (100.0%)	
Legal	4 (100.0%)	0 (0.0%)	4 (100.0%)	
Life, physical, and social science	2 (100.0%)	0 (0.0%)	2 (100.0%)	
Management	38 (100.0%)	3 (7.9%)	35 (92.1%)	
Office and administrative support	33 (100.0%)	4 (12.1%)	29 (87.9%)	
Personal care and service	35 (100.0%)	6 (17.1%)	29 (82.9%)	
Production	10 (100.0%)	1 (10.0%)	9 (90.0%)	
Protective service	33 (100.0%)	2 (6.1%)	31 (93.9%)	
Sales and related	60 (100.0%)	8 (13.3%)	52 (86.7%)	
Transportation and material moving	31 (100.0%)	4 (12.9%)	27 (87.1%)	
Customer-facing (%)				0.166
No	473 (100.0%)	51 (10.8%)	422 (89.2%)	
Yes	305 (100.0%)	43 (14.1%)	262 (85.9%)	
Work patterns (%)				0.497
No	279 (100.0%)	33 (11.8%)	246 (88.2%)	
Work from home	45 (100.0%)	8 (17.8%)	37 (82.2%)	
Yes	456 (100.0%)	54 (11.8%)	402 (88.2%)	
Days since last work (mean (SD))	4.0 (5.3)	5.0 (5.4)	3.8 (5.3)	0.159

a *Continuous variables were presented in their means and standard deviations among the population with positive and negative results, and categorical variables were presented in count and percentage. p-values were tested with independent t-test for continuous variables and were tested using χ^2^ or Fisher exact test for categorical variables. All missing values are omitted in this analysis*. *COVID-19, the Coronavirus disease 2019; SD, standard variations; RT-PCR, reverse transcriptase-polymerase chain reaction*.

There was no evident difference in the distribution of age and gender by SARS-CoV-2 assay result. Those with positive assay results were more likely to report COVID-19 exposure history (56.8 vs. 38.0%), live in a higher populated household, and reside in higher COVID-19 cumulative rate areas. Meanwhile, patients with negative results were more likely to be non-Hispanic Caucasian and current smokers. We further compared work status, job category, and work exposure between patients with positive and negative SARS-CoV-2 RT-PCR assay results. Overall, 456 of 780 (58.5%) individuals were remained at work upon presentation, and there were more HCWs in the case group (12 in 95 cases, and 51 in 685 negative individuals, *p*-value = 0.124). Meanwhile, the distribution of work patterns and the mean time since the last day at work was not different between the two groups.

### Clinical Presentations of the Study Population

Clinical characteristics among the study population were demonstrated in Table 2. The majority of the positive cases were symptomatic upon presentation (88 of 95 individuals). Patients with positive SARS-CoV-2 RT-PCR assay results had more clinical symptoms at presentations (4.3 vs. 3.4 symptoms upon the visit, *p* = 0.003). Fever/chill, cough, myalgia, and anosmia were more likely to present among positive cases than their negative counterparts.

**Table 2:** Clinical characteristics and symptoms reported by individuals in the study population during clinical intake, stratified by SARS-CoV-2 RT-PCR assay results^a^.

	**Overall**	**Positive**	**Negative**	***p*** **-value**
*N* (%)	780	95 (12.2)	685 (87.8)	
Days since onset (Mean (SD))	7.2 (10.1)	5.7 (5.2)	7.5 (10.7)	0.127
Count for symptoms (Mean (SD))	3.5 (2.5)	4.3 (2.2)	3.4 (2.6)	0.003
Symptomatic (%)	634 (100.0%)	88 (13.9%)	546 (86.1%)	0.002
Fever/chill	326 (100.0%)	60 (18.4%)	266 (81.6%)	<0.001
Headache	268 (100.0%)	41 (15.3%)	227 (84.7%)	0.054
Cough	429 (100.0%)	69 (16.1%)	360 (83.9%)	< 0.001
Shortness of breath	285 (100.0%)	30 (10.5%)	255 (89.5%)	0.284
Sore throat	302 (100.0%)	38 (12.6%)	264 (87.4%)	0.784
Myalgia	307 (100.0%)	52 (16.9%)	255 (83.1%)	0.001
Fatigue	405 (100.0%)	51 (12.6%)	354 (87.4%)	0.714
Nausea/vomiting	164 (100.0%)	20 (12.2%)	144 (87.8%)	0.995
Diarrhea	189 (100.0%)	24 (12.7%)	165 (87.3%)	0.802
Anosmia	80 (100.0%)	20 (25.0%)	60 (75.0%)	< 0.001

a *Continuous variables were presented in their means and standard deviations among the population with positive and negative results, and categorical variables were presented in counts and percentages. P-values were tested with independent t-test for continuous variables and were tested using χ^2^ or Fisher exact test for categorical variables*. *RT-PCR, reverse transcriptase-polymerase chain reaction*.

### Associations Between SARS-CoV-2 Infection and Work-Related Conditions

We conducted multivariable logistic regression to examine the association between work conditions and the likelihood of positive the SARS-CoV-2 RT-PCR assay results in Table 3. HCWs were associated with an increased odd for SARS-CoV-2 infection than those who were not working or working from home (unadjusted OR 2.30, 95% CI: 1.05–5.06; adjusted OR 4.00, 95% CI: 1.45–11.02).

**Table 3:** Associations between job families and the risk of testing positive for SARS-CoV-2 by RT-PCR assay among the study population.

**Job family**	**Unadjusted OR**	**95% CI**		**Adjusted OR** ^**a**^	**95% CI**	
Building and grounds cleaning and maintenance	1.06	0.23	4.89	0.93	0.16	5.32
Community and social service	0.80	0.23	2.76	0.61	0.11	3.32
Construction and extraction	0.43	0.10	1.87	0.23	0.03	1.92
Food preparation and serving related	1.31	0.55	3.13	2.43	0.86	6.87
Healthcare practitioners and technical	2.30	1.05	5.06	4.00	1.45	11.02
Healthcare support	0.66	0.15	2.91	0.78	0.15	3.95
Management	0.59	0.17	2.02	0.57	0.14	2.32
Office and administrative support	0.95	0.32	2.85	2.48	0.72	8.59
Personal care and service	1.43	0.56	3.65	2.28	0.76	6.85
Production	0.77	0.09	6.23	0.77	0.07	8.75
Protective service	0.45	0.10	1.94	0.75	0.14	4.13
Sales and related	1.06	0.47	2.40	1.45	0.55	3.78
Transportation and material moving	1.02	0.34	3.08	0.79	0.21	3.04

a *Adjusted for age, gender, race, smoking status, household population size, travel history, self-reported contact, and interval indicator, and the reference group included individuals that reported a work-from-home status or not currently working*.

*CI, confidence interval; OR, odds ratio; RT-PCR, reverse transcriptase-polymerase chain reaction*.

We also employed multivariable logistic regression to examine the association between job characteristics and the likelihood of positive SARS-CoV-2 RT-PCR assay (Table 4). Workers at jobs with customer-facing conditions had higher odds for SARS-CoV-2 infection compared to the rest of the population (unadjusted OR 1.36, 95% CI: 0.88–2.10; adjusted OR 1.97, 95% CI: 1.12–3.45). Meanwhile, workers who worked from home were associated with an increased likelihood of testing positive for SARS-CoV-2 than non-working individuals after adjusting for age, gender, race, smoking status, household population size, travel history, self-reported contact, and interval indicator (unadjusted OR 1.61, 95% CI: 0.69–3.76; adjusted OR 3.07, 95% CI: 1.13–8.34).

**Table 4:** Associations between customer facing, shift work, work pattern, and risk of testing positive for SARS-CoV-2 by RT-PCR assays among the study population.

**Job characteristics**	**Unadjusted OR**	**95% CI**		**Adjusted OR** ^**a**^	**95% CI**	
Customer-facing	1.36	0.88	2.10	1.97	1.12	3.45
Shift work	1.29	0.79	2.09	1.63	0.91	2.94
Work pattern^b^						
Work from home	1.61	0.69	3.76	3.07	1.13	8.34
In person	1.00	0.63	1.59	1.47	0.80	2.69

a *Adjusted for age, gender, race, smoking status, household population size, travel history, self-reported contact, and interval indicator*.

b *The reference group was individuals with self-reported non-working status*.

*CI, confidence interval; OR, odds ratio; RT-PCR, reverse transcriptase-polymerase chain reaction*.

### Sensitivity Analysis

The multivariable regression model showed associations for contact history, and race, and decreased risk for smoking after phase I reopening (Supplementary Table 1). Meanwhile, the associations were similar after restricting the analysis to individuals tested prior to phased reopening (Supplementary Tables 2, 3). The associations for HCWs remained significant after using inverse probability weighting to balance the distribution of covariates, and we did not identify other evident associations for other job families (Supplementary Table 4). We found that individuals reporting work status as in-person were more likely to report exposure to suspected/confirmed COVID-19 customers or colleagues, and they were more likely to have a shorter interval between symptom onset and clinic visit than those who were not working or working from home (Supplementary Tables 5, 6). Lastly, we examined the demographic and clinical presentations for patients tested positive by SARS-CoV-2 RT-PCR assay and worked from home. These patients were mostly diagnosed in the first month of the study, and three out of eight subjects reported COVID-19 exposure history with their families (Table 5).

**Table 5:** Descriptions of detailed (a) demographics and (b) reported clinical symptoms of individuals with positive SARS-CoV-2 RT-PCR assay result who reported they worked from home during the initial intake evaluation.

**a. Demographic conditions**											
**No**	**Encounter Date**	**Age**	**Sex**	**Race**	**Job family**	**Travel history**	**Exposure source**	**Cohabitant number**	**Smoker**	**Days since symptom onset**	
1	3/19/2020	49	Female	Black or African American	Educational instruction and library	No	No	1	No	1	
2	3/26/2020	55	Male	White	Business and financial operations	No	No	0	Yes	7	
3	4/1/2020	53	Female	White	Healthcare support	No	Families	1	No	1	
4	4/2/2020	32	Female	Asian	Computer and mathematical	No	No	3	No	6	
5	4/11/2020	33	Male	White	Educational instruction and library	No	Families	1	Yes	4	
6	4/14/2020	28	Female	Black or African American	Arts, design, entertainment, sports, and media	No	Families	4	No	6	
7	4/20/2020	36	Female	Asian	Business and financial operations	No	Colleagues	1	No	8	
8	5/8/2020	35	Female	Asian	Transportation and material moving	No	No	4	No	2	
**b. Clinical symptoms**											
**No**	**Fever/chills**	**Headache**	**Cough**	**Shortness of breath**	**Sore throat**	**Myalgia**	**Fatigue**	**Nausea**	**Diarrhea**	**Anosmia**	**Other symptoms**
1	Yes	No	Yes	No	Yes	No	No	No	No	No	No
2	Yes	No	Yes	No	Yes	Yes	Yes	No	Yes	Yes	No
3	Yes	Yes	Yes	Yes	No	Yes	Yes	No	Yes	No	No
4	Yes	Yes	Yes	No	Yes	Yes	Yes	Yes	Yes	Yes	Vomiting, ageusia, nasal congestion, eye pain, sinus pressure
5	No	Yes	Yes	No	Yes	Yes	Yes	No	No	No	Sneezing, sinus pressure
6	Yes	Yes	Yes	Yes	Yes	Yes	No	Yes	No	No	Wheezing
7	Yes	No	Yes	No	Yes	Yes	No	No	No	No	Nasal congestion
8	Yes	Yes	Yes	No	Yes	No	No	No	No	No	No

*RT-PCR, Reverse transcription polymerase chain reaction*.

## Discussion

Several occupation-related risk factors resulting in a positive SARS-CoV-2 assay result were identified in this cohort of community residents in the U.S. To begin with, healthcare workers were 4 times more likely to have a positive SARS-CoV-2 assay result. While not statistically significant, we also observed an increased risk among workers in the food preparation, office administration, and personal care professions. Furthermore, individuals with customer-facing jobs had a two-fold risk increase in testing positive on the SARS-CoV-2 assay. Individuals working from home were associated with a higher likelihood of testing positive for SARS-CoV-2 at the earlier phase (unadjusted OR 1.61, 95% CI: 0.69–3.76; adjusted OR 3.07, 95% CI: 1.13–8.34). Additionally, individuals with a positive SARS-CoV-2 assay result were more likely to live in households with higher resident counts, in communities with higher cumulative incidence rates, and/or reported COVID-19 exposure with family or friends. To the best of our knowledge, this study is the first to demonstrate these associations between an individual's occupation, customer exposure through jobs, and SARS-CoV-2 assay results in a cohort of community residents in the U.S.

The increased risks among healthcare workers were consistently observed in multiple analyses throughout this study, which is in concordance with results observed in previous studies (23–25, 40–42). At the same time, previous studies that observed similar presenting symptoms and/or elevated SARS-CoV-2 positivity risks were conducted among healthcare workers in hospital-based settings (23, 25). Our study examined the risk among HCWs from different healthcare facilities and settings in a community-based cohort, which extended the scope from previously published hospital-based, single-setting studies. Additionally, a panel of occupational medicine physicians reviewed and verified each HCW's job title and work-related exposure under a standardized protocol. This rigorous approach provides a more granular information for individual's occupation and work status, extending the HCW occupational risk findings and associations previously identified in studies that utilized aggregated U.S. and U.K. databases (24, 43).

In addition to healthcare workers, we identified increased odds of having a positive SARS-CoV-2 assay result among workers in customer-facing roles and those who reported they worked from home. Individuals with customer-facing jobs had a two-fold increase in risk of being tested positive for SARS-CoV-2. This finding we observed among individuals with customer facing jobs may be associated with the increased risk of direct exposure to coronavirus infected customers at workplace (12, 17, 19, 33, 36). In a previous study summarizing work-related COVID-19 cases in six Asian countries, it was hypothesized that these workers contracted COVID-19 through contact exposure to their customers (19). Another study among retail workers in Massachusetts also identified an increased risk in testing positive for SARS-CoV-2 in store employees with customer-facing roles (12). In further examining specific job categories, we observed an increased risk of SARS-CoV-2 positivity among workers in the food preparation, office admin, and personal care job categories, albeit the increase was not statistically significant among our cohort. At the same time, this study provided detailed occupation information on the population at risk, which filled in the scientific gap in the limitation of previous research using aggregated information from the Workers' compensation database (27).

Surprisingly, in this study we observed an increased risk in having a positive SARS-CoV-2 assay result among workers who reported a work-from-home status at the time of COVID-19 exposure or symptom onset compared to those who were not at work. This finding may be due to household clustering, as three of the eight positive cases in the work-from-home group reported exposure to confirmed COVID-19 household contacts. Additionally, household population and exposure to confirmed COVID-19 family members were associated with increased risk of SARS-CoV-2 assay positivity in this study. This may be due to shared spaces (4, 5), frequent interaction with infectious individuals at home (3, 6, 35, 37), or less adherence to maintaining social distancing within a more congregated household (18, 34, 38, 44). Therefore, the high proportion of reported household transmission among these work-from-home workers provided a possible explanation for the increased likelihood of SARS-CoV-2 infection we observed in this study, as work-from-home individuals are less likely to wear personal protective equipment at home and may have significant exposure to SARS-CoV-2 from their infected family members (4, 34–36, 38). Additionally, lengthened work hours and increased occupational stress due to workplace transition among work-from-home workers during this first wave of the pandemic may have further increased their susceptibility to SARS-CoV-2 (45). Lastly, as we observed a wide confidence interval for the estimate, the possibility of unmeasured confounders and temporal ambiguity cannot be ruled out.

There are several strengths to this study. First, the job category, customer exposure and work status of each patient was examined and classified independently by three occupational medicine physicians in a rigorous, blinded approach as the evaluators were unaware of SARS-CoV-2 testing results during the classification process. The results have also been validated internally for test-retest consistency to provide a more accurate and granular information of an individual's occupational status. Our approach and study results filled in the knowledge gap of previous studies that used public health databases, as those studies do not have the detailed work history as we collected in this study. Second, data were collected by multiple experienced licensed clinicians before testing in a preset, templated format, which minimized information and recall bias. Third, the nasopharyngeal SARS-CoV-2 RT-PCR test was utilized in all patients in this study, which is among the most widely used and accurate testing methods for SARS-CoV-2 detection (32). Last not the least, we adjusted for personal risk factors in this community-based population to reduce the confounding from individual factors.

There are several limitations to this study as well. First, there were unmeasured socioeconomic status confounders, such as family income and education level, which may lead to non-differential bias. Second, while we utilized templated intake questions with clear questions and answer choices conducted by licensed clinicians, there is a chance that individuals may have mistakenly reported their work status or exposure history. These misclassifications are non-differential under the cohort design, but they may bias the results toward the null. Third, while we included a moderate cohort size in this study, the extensive job category list led to wider confidence intervals and less power to detect smaller differences. Therefore, we were not able to distinguish the differences between frontline and supporting healthcare workers, and there was a wide confidence interval for the association on shift workers. Lastly, this study included individual data from the first wave of the pandemic, with the Massachusetts state of emergency and the Order to shutdown non-essential services, we were only able to capture essential workers' work-related exposure risks during the first wave and the subsequent initial phase of reopening. Additionally, the Massachusetts testing guideline excluded asymptomatic individuals from obtaining a SARS-CoV-2 test during this period of the pandemic. With the increase in population immunity from both COVID-19 vaccine and natural infection, the results from our study therefore cannot be fully generalized to our present state in this pandemic. At the same time, this limitation caused by the state non-essential services shutdown order and the strict testing criteria created a unique environment with less confounders and allowed us to specifically examine the workplace exposure risks for non-HCW essential workers at the onset of this pandemic, providing valuable insights and lessons to workplace communicable disease emergency response planning for essential services that can be used for the future.

In conclusion, this study identified several significant correlations between individuals' occupational exposure and risk of SARS-CoV-2 infection during the first wave of the COVID-19 pandemic. Our study demonstrated a four-time increased risk of SARS-CoV-2 assay positivity among healthcare workers. Moreover, workers with customer-facing jobs were associated with a two-fold increased risk in testing positive for SARS-CoV-2, suggesting a higher COVID-19 occupational risk for workplaces with direct, face-to-face customer exposures. While further research is warranted to determine if the observed correlations continued in this current state of the pandemic due to population immunity and natural attenuation of SARS-CoV-2 virulence, correlations observed in this study for non-healthcare essential workers provide significant insights for workplace communicable disease emergency response planning in the future.

## Data Availability Statement

The raw data supporting the conclusions of this article will be made available by the authors, without undue reservation.

## Ethics Statement

The studies involving human participants were reviewed and approved by Institutional Review Board of Boston University (IRB H-40496). Written informed consent to participate in this study was provided by the participant, or participants' legal guardian/next of kin.

## Author Contributions

C-FW, F-YL, Y-TH, and JY contributed to conception and design of the study and wrote sections of the manuscript. NL, LD, RA, and JY organized the database. C-FW performed the statistical analysis and wrote the first draft of the manuscript. All authors contributed to manuscript revision, read, and approved the submitted version.

## Conflict of Interest

The authors declare that the research was conducted in the absence of any commercial or financial relationships that could be construed as a potential conflict of interest.

## Publisher's Note

All claims expressed in this article are solely those of the authors and do not necessarily represent those of their affiliated organizations, or those of the publisher, the editors and the reviewers. Any product that may be evaluated in this article, or claim that may be made by its manufacturer, is not guaranteed or endorsed by the publisher.
