# Supplementary material for: Risk of SARS-CoV-2 Infection Among Essential Workers in a Community-Based Cohort in the United States
Source: Front Public Health. 2022 May 17;10:878208. doi: 10.3389/fpubh.2022.878208 (PMC9169416; doi:10.3389/fpubh.2022.878208)
Supplement: Supplementary file 1 [file Table_1.DOCX]

| **Supplement Table 1.** *Multivariable logistic regression for the adjusted odds ratio and 95% confidence interval estimates for the association between included covariates and SARS-CoV-2 RT-PCR positive assay results among study population ^a^* | | | | |
| --- | --- | --- | --- | --- |
| Covariates | Adjusted OR |  | 95% CI | |
| Age | 1.00 | 0.98 | | 1.02 |
| Gender as male | 1.15 | 0.70 | | 1.89 |
| Race |  |  | |  |
| Black | 3.77 | 1.79 | | 7.91 |
| Asian | 2.56 | 1.21 | | 5.40 |
| Hispanic | 3.20 | 1.36 | | 7.50 |
| Others | 2.77 | 1.37 | | 5.61 |
| Smoking: Yes | 0.27 | 0.12 | | 0.61 |
| Household population size | 1.12 | 0.97 | | 1.29 |
| Travel history: Yes |  |  | |  |
| Interval indicator |  |  | |  |
| Extended criteria | 0.61 | 0.35 | | 1.09 |
| After re-open ^b^ | 0.27 | 0.13 | | 0.54 |

1. Estimates were adjusted for other covariates listed in this Table.
2. Reopening is defined as the period between the start date of the Massachusetts state non-essential services shutdown order to the start date of the phase 1 reopening, between Mar 18^th^, 2020, to May 18^th^, 2020.

Abbreviations: CI: confidence interval, OR: odds ratio

| **Supplement Table 2.** *Multivariable logistic regression for the associations between job families and SARS-CoV-2 RT-PCR positive assay results among study population during the initial Massachusetts non-essential services shutdown period to phase 1 reopening ^a^* (n=614) | | | |
| --- | --- | --- | --- |
| Job family | Adjusted OR ^b^ | 95% CI | |
| Building and Grounds Cleaning and Maintenance | 0.93 | 0.16 | 5.32 |
| Community and Social Service | 0.61 | 0.11 | 3.32 |
| Construction and Extraction | 0.23 | 0.03 | 1.92 |
| Food Preparation and Serving Related | 2.43 | 0.86 | 6.87 |
| Healthcare Practitioners and Technical | 4.00 | 1.45 | 11.02 |
| Healthcare Support | 0.78 | 0.15 | 3.95 |
| Management | 0.57 | 0.14 | 2.32 |
| Office and Administrative Support | 2.48 | 0.72 | 8.59 |
| Personal Care and Service | 2.28 | 0.76 | 6.85 |
| Production | 0.77 | 0.07 | 8.75 |
| Protective Service | 0.75 | 0.14 | 4.13 |
| Sales and Related | 1.45 | 0.55 | 3.78 |
| Transportation and Material Moving | 0.79 | 0.21 | 3.04 |
| 1. Reopening is defined as the period between the start date of the Massachusetts state non-essential services shutdown order to the start date of the phase 1 reopening, between Mar 18^th^, 2020, to May 18^th^, 2020. 2. Adjusted for age, gender, race, smoking status, household population size, travel history, self-reported contact, and interval indicator.   Abbreviations: CI: confidence interval, OR: odds ratio, RT-PCR: reverse transcriptase-polymerase chain reaction | | | |
|  | | | |

| **Supplement Table 3.** *Multivariable logistic regression for the associations between job conditions and SARS-CoV-2 RT-PCR positive assay results among study population during the initial Massachusetts non-essential services shutdown period to phase 1 reopening ^a^ (n=614)* | | | |
| --- | --- | --- | --- |
| Job conditions | Adjusted OR ^b^ | 95% CI | |
| Customer facing | 1.65 | 0.90 | 3.03 |
| Self-reported COVID-19 exposure source ^c^ |  |  |  |
| *Family or friends* | 3.75 | 1.92 | 7.31 |
| *Colleagues or customers* | 1.15 | 0.56 | 2.34 |
| Current work status |  |  |  |
| Work from home | 4.53 | 1.49 | 13.75 |
| At work | 1.54 | 0.78 | 3.04 |
| \| a. Reopening is defined as the period between the start date of the Massachusetts state non-essential services shutdown order to the start date of the phase 1 reopening, between Mar 18^th^, 2020, to May 18^th^, 2020.  b. Adjusted for age, gender, race, smoking status, household population size, self-reported contact, and interval indicator. \| \| --- \| \| c. Adjusted for age, gender, race, smoking status, household population size and interval indicator. Abbreviations: CI: confidence interval, OR: odds ratio, RT-PCR: reverse transcriptase-polymerase chain reaction \| | | | |

| **Supplement Table 4.** *Multivariable logistic regression for the associations between job conditions and SARS-CoV-2 RT-PCR positive assay results among study population with inverse probability weighting* | | | |
| --- | --- | --- | --- |
| Job family | Adjusted OR after IP weighting ^a, b^ | 95% CI | |
| Building and Grounds Cleaning and Maintenance | 2.06 | 0.19 | 21.97 |
| Community and Social Service | 0.24 | 0.02 | 3.70 |
| Construction and Extraction | 0.12 | 0.01 | 2.46 |
| Food Preparation and Serving Related | 2.92 | 0.86 | 9.92 |
| Healthcare Practitioners and Technical | 15.57 | 4.52 | 53.56 |
| Healthcare Support | 0.49 | 0.04 | 5.83 |
| Management | 0.44 | 0.07 | 2.81 |
| Office and Administrative Support | 1.17 | 0.14 | 9.94 |
| Personal Care and Service | 1.98 | 0.50 | 7.74 |
| Production | 0.36 | 0.02 | 6.09 |
| Protective Service | 0.38 | 0.05 | 3.26 |
| Sales and Related | 0.47 | 0.11 | 2.09 |
| Transportation and Material Moving | 0.46 | 0.07 | 3.04 |
| a. Adjusted for age, gender, race, smoking status, household population size, travel history, self-reported contact, and interval indicator. | | | |
| b. Inverse probability weighted with symptoms upon visiting.  Abbreviations: CI: confidence interval, OR: odds ratio, RT-PCR: reverse transcriptase-polymerase chain reaction | | | |

| **Supplement Table 5.** *Baseline demographics for the study population*, *stratified by work status at their presence* | | | | |
| --- | --- | --- | --- | --- |
|  | Not at work | Work from home | Working | p-value ^a^ |
| N (%) | 279 (35.8) | 45 (5.8) | 456 (58.5) |  |
| Age (mean (SD)) | 42.2 (13.5) | 42.6 (12.6) | 41.8 (12.2) | 0.852 |
| Gender (%) |  |  |  | 0.012 |
| Female | 176 (63.1) | 31 (68.9) | 236 (51.8) |  |
| Male | 103 (36.9) | 14 (31.1) | 218 (47.8) |  |
| NA | 0 (0.0) | 0 (0.0) | 2 (0.4) |  |
| Race (%) |  |  |  | 0.176 |
| Non-Hispanic white | 150 (53.8) | 30 (66.7) | 263 (57.7) |  |
| Black | 18 (6.5) | 4 (8.9) | 34 (7.5) |  |
| Asian | 30 (10.8) | 6 (13.3) | 41 (9.0) |  |
| Hispanics | 19 (6.8) | 0 (0.0) | 25 (5.5) |  |
| Others | 36 (12.9) | 1 (2.2) | 38 (8.3) |  |
| NA | 26 (9.3) | 4 (8.9) | 55 (12.1) |  |
| Smoking (%) |  |  |  | 0.178 |
| None | 203 (72.8) | 40 (88.9) | 346 (75.9) |  |
| Yes | 76 (27.2) | 5 (11.1) | 109 (23.9) |  |
| NA | 0 (0.0) | 0 (0.0) | 1 (0.2) |  |
| Household population size (mean (SD)) | 3.3 (1.9) | 2.7 (1.6) | 3.0 (1.7) | 0.020 |
| Self-reported COVID-19 exposure source (%) |  |  |  | <0.001 |
| None | 195 (69.9) | 31 (68.9) | 238 (52.2) |  |
| Family/Friend | 78 (28.0) | 10 (22.2) | 59 (12.9) |  |
| Colleague/Customer | 4 (1.4) | 4 (8.9) | 158 (34.6) |  |
| NA | 2 (0.7) | 0 (0.0) | 1 (0.2) |  |
| Travel history (%) |  |  |  | 0.014 |
| None | 262 (93.9) | 38 (84.4) | 434 (95.2) |  |
| Yes | 15 (5.4) | 7 (15.6) | 22 (4.8) |  |
| NA | 2 (0.7) | 0 (0.0) | 0 (0.0) |  |
| Customer facing (%) |  |  |  | <0.001 |
| None | 277 (99.3) | 41 (91.1) | 155 (34.0) |  |
| Yes | 0 (0.0) | 4 (8.9) | 301 (66.0) |  |
| NA | 2 (0.7) | 0 (0.0) | 0 (0.0) |  |
| Last day at work (mean (SD)) | 27.0 (10.5) | 2.0 (3.6) | 3.5 (3.8) | <0.001 |

1. Continuous variables were presented in their means and standard deviations among the population who were not at work, working from home and working, and categorical variables were presented in counts and percentages. P-values were tested with independent t-test for continuous variables and were tested using χ^2^ or Fisher exact test for categorical variables.

Abbreviations: SD: standard deviation, NA: not available

| **Supplement Table 6.** *Clinical characteristics and symptoms reported by individuals in the study population during clinical intake, stratified by work status at presence* | | | | |
| --- | --- | --- | --- | --- |
|  | Not at work | Work from home | Working | p-value ^a^ |
| N | 279 | 45 | 456 |  |
| Positive cases (%) | 33 (11.8) | 8 (17.8) | 54 (11.8) | 0.497 |
| Days since onset (mean (SD)) | 9.6 (13.6) | 9.6 (12.2) | 5.6 (6.3) | <0.001 |
| Count of symptoms at presence (mean (SD)) | 3.6 (2.7) | 3.6 (2.7) | 3.5 (2.4) | 0.777 |
| Symptom (%) | 227 (81.4) | 37 (82.2) | 370 (81.1) | 0.983 |
| Fever/chill | 115 (41.2) | 22 (48.9) | 189 (41.4) | 0.609 |
| Headache | 93 (33.3) | 21 (46.7) | 154 (33.8) | 0.200 |
| Cough | 159 (57.0) | 27 (60.0) | 243 (53.3) | 0.487 |
| Shortness of breath | 108 (38.7) | 16 (35.6) | 161 (35.3) | 0.643 |
| Sore throat | 108 (38.7) | 20 (44.4) | 174 (38.2) | 0.711 |
| Myalgia | 112 (40.1) | 14 (31.1) | 181 (39.7) | 0.503 |
| Fatigue | 143 (51.3) | 22 (48.9) | 240 (52.6) | 0.857 |
| Nausea/vomiting | 72 (25.8) | 5 (11.1) | 87 (19.1) | 0.023 |
| Diarrhea | 62 (22.2) | 11 (24.4) | 116 (25.4) | 0.614 |
| Anosmia | 36 (12.9) | 3 (6.7) | 41 (9.0) | 0.170 |
| 1. Continuous variables were presented in their means and standard deviations among the population who were not at work, working from home and working, and categorical variables were presented in counts and percentages. P-values were tested with independent t-test for continuous variables and were tested using χ^2^ or Fisher exact test for categorical variables.   Abbreviations: SD: standard deviation, NA: not available | | | | |
